# Antibacterial silver ion-coated dental implants suppress peri-implantitis in a murine model

**DOI:** 10.1038/s41598-026-53121-2

**Published:** 2026-05-14

**Authors:** Mana Nasu, Tomoya Soma, Hidetaka Miyashita, Takehito Ouchi, Yoshitaka Kase, Takazumi Yasui, Fuka Homma, Kitaru Suzuki, Takeshi Miyamoto, Hideyuki Okano, Masaya Nakamura, Taneaki Nakagawa, Mamoru Aizawa, Satoru Morikawa

**Affiliations:** 1 Department of Dentistry and Oral Surgery, Keio University School of Medicine, 35 Shinanomachi, Shinjuku-ku, Tokyo, 160-8582 Japan; 2 Department of Physiology, Tokyo Dental College, 2-9-18, Kanda-Misaki-cho, Chiyoda-ku, Tokyo, 101-0061 Japan; 3 Division of CNS Regeneration and Drug Discovery, International Center for Brain Science (ICBS), Fujita Health University, 1-98 Dengakugakubo, Kutsukake-cho, Toyoake-shi, Aichi 470-1192 Japan; 4 Regenerative Medicine Research Center, Keio University, 3-25-10 Tonomachi, Kawasaki-ku, Kawasaki-shi, Kanagawa 210-0821 Japan; 5 Department of Materials Science and Technology Faculty of Advanced Engineering, Tokyo University of Science, 6-3-1 Niijuku, Katsushika-ku, Tokyo, 125-8585 Japan; 6 Department of Orthopedic Surgery, Kumamoto University, 1-1-1 Honjo, Chuo-ku, Kumamoto, 860-8556 Japan; 7 Department of Orthopaedic Surgery, Keio University School of Medicine, 35 Shinanomachi, Shinjuku-ku, Tokyo, 160-8582 Japan; 8 Department of Applied Chemistry, School of Science and Technology, Meiji University, 1-1-1 Higashimita, Tama-ku, Kawasaki, Kanagawa 214-8571 Japan

**Keywords:** Antibacterial coating, Dental implant, Peri-implantitis, *Porphyromonas gingivalis*, Silver ion, Biotechnology, Diseases, Health care, Medical research, Microbiology

## Abstract

Peri-implantitis, an inflammatory condition caused by bacterial infection around an implant, is currently the leading cause of implant failure. *Porphyromonas gingivalis* (*P. gingivalis*), an anaerobic bacterial pathogen associated with periodontitis, is known to play a key role in peri-implantitis. To address this issue, the present study examined the antibacterial properties of silver ion (Ag^+^)-coated titanium implants against *P. gingivalis* and their ability to prevent bone loss. Ag^+^-coated implants, i.e., Ti implants coated with Ag^+^ ions on a hydroxyapatite film chelated with inositol hexaphosphate, demonstrated significant antibacterial activity against *P. gingivalis* in the Ti wire configuration in inhibition zone assays (*n* = 4 per group). Furthermore, in a murine model of ligature-induced peri-implantitis, these implants significantly reduced alveolar bone resorption compared to uncoated titanium controls. This preclinical study suggests that applying an Ag^+^ coating to dental implants is an effective strategy for preventing *P. gingivalis*–induced peri-implantitis. In the control group, bone loss of 19–25% relative to baseline was observed at day 28, whereas the Ag^+^-coated group exhibited only 15–20% bone loss (*n* = 8 per group per time point). These findings suggest the potential of Ag^+^ coating as a preventive strategy against peri-implantitis-associated bone loss.

## Introduction

Dental implants are the preferred solution for replacing missing teeth and effectively restoring function and aesthetics^1^. The long-term success of these implants relies on osseointegration—the formation of a direct structural and functional bond with the adjacent bone^2^. However, despite their advantages, implants are susceptible to bacterial infections that can lead to peri-implant diseases, namely peri-implant mucositis and the more destructive peri-implantitis^3^. Such infections can emerge at any stage, even years after the placement of the prosthetic superstructure^4^.

Peri-implantitis, a bacteria-induced inflammatory disease affecting implant-supporting tissues, is a major cause of dental implant failure^5^. With a high prevalence reported in the 20%–47% range^6^, its prevention and management are clinical priorities. Furthermore, the condition is complicated by shared pathogenic mechanisms with periodontitis, which has been linked to systemic diseases such as Alzheimer’s disease^7^ and cardiovascular disorders^8^.

Failure of implant-supported restorations is frequently attributed to peri-implantitis^9,10^. Conventional treatments, such as mechanical debridement, are often insufficient to resolve the condition due to complex implant topographies and irregular defects. Additionally, reliance on repeated antibiotic use has contributed to the growing problem of bacterial resistance^11^. This challenge is compounded by findings from randomized controlled trials, which show that systemic antimicrobial agents lack efficacy in treating peri-implantitis^12,13^, highlighting the need for novel strategies to combat biofilm infections^14^.

The development of implants with inherent antimicrobial properties is a promising strategy to address the limitations of current therapies. However, it is crucial that these antimicrobial features do not compromise the structural integrity or biocompatibility of implants. Among various approaches^15^, coating implants with silver ions (Ag^+^) has emerged as particularly effective. Previous studies have shown that Ag^+^ effectively inhibits pathogens such as *Staphylococcus aureus* and suppresses biofilm formation without causing cytotoxicity^16,17^. These antibacterial effects are often attributed to the generation of reactive oxygen species in aerobic environments^18,19^; however, the precise mechanisms remain incompletely understood.

The anaerobic bacterium *Porphyromonas gingivalis* (*P. gingivalis*), a key pathogen in periodontitis, is also implicated in peri-implantitis and subsequent implant failure^20–22^. Although Ag^+^ has been shown to be effective against aerobic pathogens through oxidative stress mechanisms, its antimicrobial efficacy under anaerobic conditions—particularly against *P. gingivalis*—has remained unexplored. Among the pro-inflammatory cytokines implicated in peri-implantitis, interleukin-1β (IL-1β), interleukin-6 (IL-6), and tumor necrosis factor-alpha (TNFα) are central mediators of inflammatory tissue destruction. Elevated levels of these cytokines have been consistently reported in peri-implant crevicular fluid from affected sites and correlate with disease severity^23–25^. Therefore, these cytokines were selected as molecular markers to evaluate the anti-inflammatory effect of the Ag^+^ coating in the present study. This gap underscores the necessity of evaluating Ag^+^-coated implants particularly within anaerobic peri-implantitis models.

We hypothesized that Ag^+^ coating on titanium implants would exert antibacterial activity against *P. gingivalis* under anaerobic conditions and attenuate peri-implant bone loss. The primary objective of this study was to evaluate the in vitro antibacterial efficacy of Ag^+^-coated Ti implants against *P. gingivalis*. The secondary objective was to assess the in vivo protective effect of Ag^+^-coated implants on peri-implant bone levels and inflammatory cytokine expression in a murine ligature-induced peri-implantitis model.

## Results

### Inhibition zone assay

**Fig. 1:**
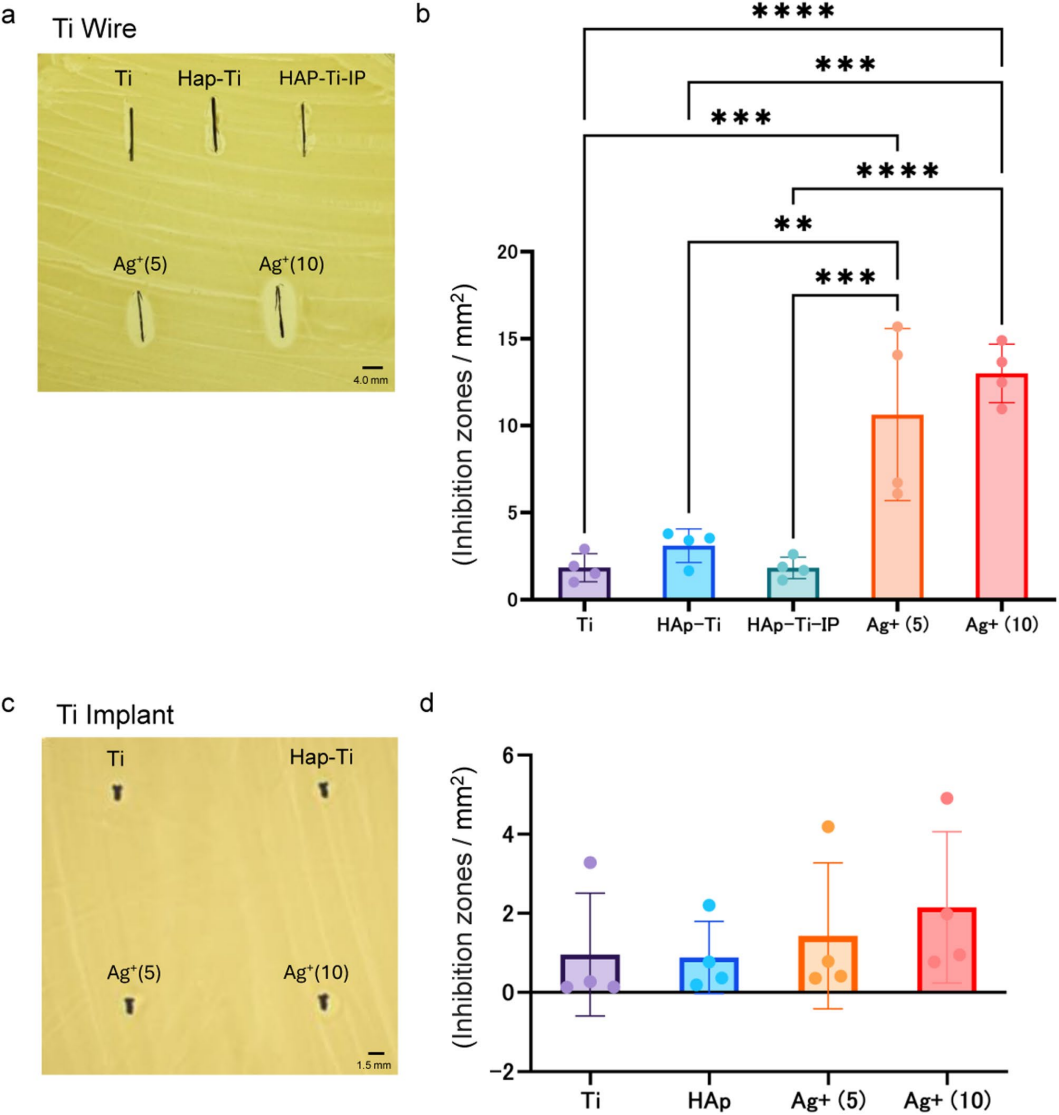
In vitro antibacterial activity of the Ag^+^-coated materials against *P. gingivalis*. (**a**) Inhibition zones formed by five groups of Ti wires (0.5 mm width × 8 mm length): (1) unmodified Ti, (2) HAp-coated, (3) IP6-modified HAp-coated, (4) HAp-IP6-Ag^+^ (5 mM), and (5) HAp-IP6-Ag^+^ (10 mM). (**b**) Quantitative analysis of inhibition zones for the Ti wire experiment. (**c**) Inhibition zones formed by four groups of Ti implant screws (0.8 mm diameter × 1.5 mm length): (1) unmodified Ti, (2) HAp-coated, (3) HAp-IP6-Ag^+^ (5 mM), and (4) HAp-IP6-Ag^+^ (10 mM). (**d**) Quantitative analysis of inhibition zones for the implant screws. Data are presented as mean ± standard deviation (*n* = 4). Asterisks indicate statistical significance (*p < 0.05, **p < 0.01, ***p < 0.001, ****p < 0.0001); n.s., not significant (one-way ANOVA followed by Tukey’s post-hoc test).

The antibacterial activity of Ag^+^-coated implants against *P. gingivalis* was evaluated using an inhibition zone assay, with quantitative data presented in Table 1. Five specimen groups were tested in the Ti wire experiments: unmodified Ti; hydroxyapatite (HAp); inositol hexaphosphate (IP6)-modified HAp-coated; and HAp-IP6-Ag^+^ at two concentrations [Ag^+^(5) and Ag^+^(10)]. Only the HAp-IP6-Ag^+^ Ti wires exhibited antibacterial activity, forming clear inhibition zones (Fig. 1a). While inhibition zone size tended to increase with higher Ag^+^ concentrations, the difference between Ag^+^(5) and Ag^+^(10) did not reach statistical significance (Fig. 1b).A parallel experiment was conducted using four groups of implant screws (Fig. 1c). This assay produced smaller but still detectable inhibition zones; however, no statistically significant differences were observed among the groups (Fig. 1d). Nonetheless, the Cohen’s d between uncoated implants and Ag^+^(10)-coated implants was 0.43, indicating a small effect size, which suggests a trend toward antibacterial activity in the Ag^+^-coated implant screws.

**Table 1:** Quantitative data for the inhibition zone assay. Mean, standard deviation (SD), and median size of the inhibition zone values are shown for both the (a) Ti wire and (b) Ti implant screw experiments.

a			
Ti wire	Sample number (N)	Mean ± SD (mm^2^)	Median (mm^2^)
Ti	4	1.84 ± 0.81	1.71
HAp	4	3.10 ± 0.97	3.46
HAp-IP	4	1.82 ± 0.61	1.78
Ag^+^ (10)	4	13.00 ± 1.68	13.07
Ag^+^ (5)	4	10.64 ± 4.94	10.39

b			
Ti implant	Sample number (*N*)	Mean ± SD (mm^2^)	Median (mm^2^)
Ti	4	0.96 ± 1.55	0.21
HAp	4	0.88 ± 0.91	0.57
Ag^+^ (10)	4	1.43 ± 1.85	0.59
Ag^+^ (5)	4	2.15 ± 1.91	1.47

### Clinical observations

In the initial phase, most surgical sites healed uneventfully following tooth extraction and implant placement. Initial stability was confirmed for all implants at the time of placement; however, four implants were lost over the course of the experimental period. No visible inflammation was observed before or immediately after ligature placement. By day 28 post-ligation, control implants began to exhibit clear signs of peri-implant inflammation, including gingival erythema and, in some cases, mobility. In contrast, Ag^+^-coated implants remained clinically stable throughout the experiment.

### Radiographic analysis

**Fig. 2:**
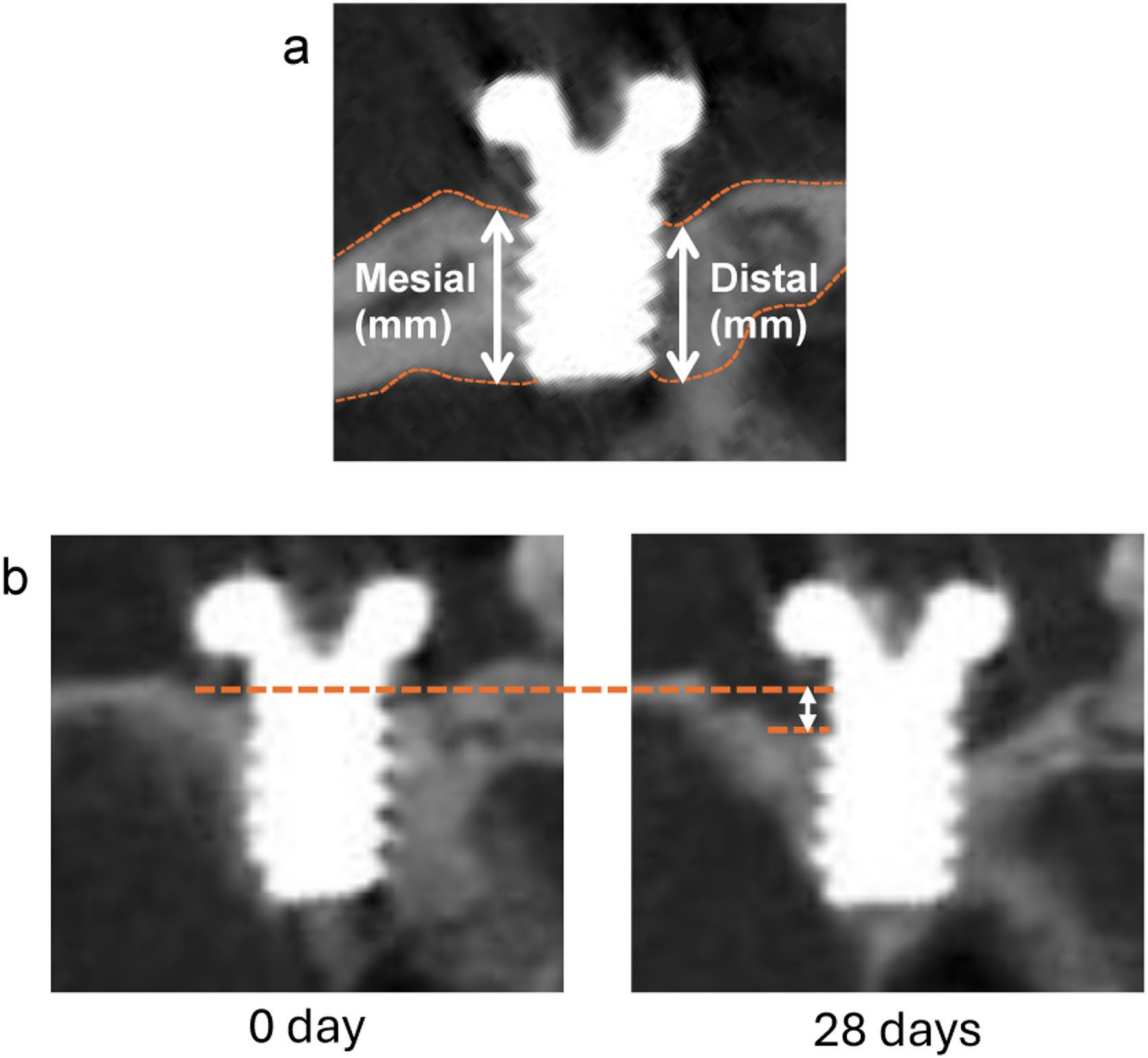
Schematic of peri-implant bone-level measurements. (**a**) White double-headed arrows indicate the peri-implant bone level, defined as the vertical distance from the alveolar bone crest (ABC) to the apex of the implant (i.e., the length of the implant embedded within bone), at the mesial (M) and distal (D) sites. These measurements were used to calculate the percentage of bone loss relative to the baseline. (**b**) Determination of bone loss. The white double-headed arrows indicate the difference in vertical bone level around the implant, quantitatively analyzed as the difference in peri-implant bone level between day 0 and day 28.

Changes in peri-implant bone levels following ligature placement were assessed using micro-computed tomography (CT). The peri-implant bone level, defined as the vertical distance from the alveolar bone crest (ABC) to the apex of the implant (i.e., the length of the implant embedded within bone), was measured at the mesial and distal sites (Fig. 2), and these values were used to calculate bone loss as a percentage relative to baseline (pre-ligation) measurements.

**Fig. 3:**
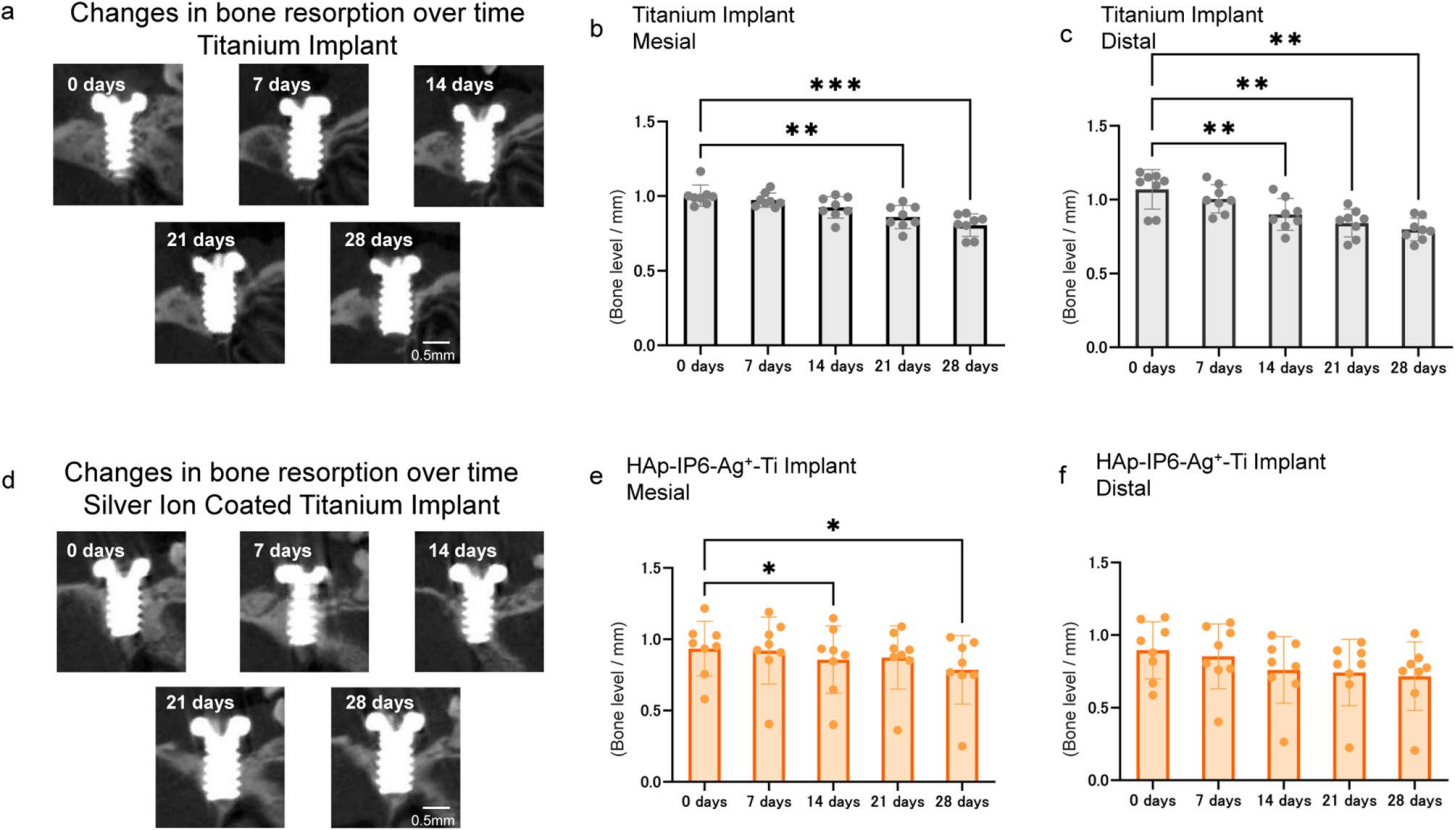
Micro-CT analysis of time-dependent changes in peri-implant bone resorption. (**a**,**d**) Representative sagittal micro-CT images of an uncoated (**a**) and Ag^+^-coated (**d**) implant at 0, 7, 14, 21, and 28 days post-ligation. (**b**,**c**) Quantitative bone levels at the (**b**) mesial and (**c**) distal sites in the control group. (**e**,**f**) Bone levels at the (**e**) mesial and (**f**) distal sites in the Ag^+^-coated group. Data are shown as mean ± standard deviation (*n* = 8). Asterisks indicate statistical significance: *p < 0.05, **p < 0.01, ***p < 0.001; comparisons were made between baseline (day 0) and each subsequent time point (repeated measures one-way ANOVA followed by Dunnett’s post-hoc test).

In the control group, baseline bone levels (day 0) were 1.00 ± 0.07 mm (mesial) and 1.07 ± 0.13 mm (distal), respectively (Table 2). These values were shorter than the 1.5-mm implant length due to supramucosal placement, which allowed sufficient space for ligature attachment. At the mesial site, significant bone resorption was not observed until day 14 but progressed from day 21 onward, consistent with established peri-implantitis models. No significant difference in bone levels was noted between days 21 and 28 (Fig. 3a, b). Conversely, at the distal site, significant bone resorption was observed beginning on day 7 (Fig. 3a, c). By day 28, control implants showed 19%–25% bone loss relative to baseline, with final bone levels measuring 0.81 ± 0.08 mm (mesial) and 0.80 ± 0.077 mm (distal). No significant difference was detected between the mesial and distal sites.

**Table 2:** Comparison of peri-implant bone levels before (day 0) and after (day 28) ligature placement in the control and Ag^+^-coated implant groups.

Normal	Sample number (N)	Bone level before ligature (mm)		Bone level after ligature (day 28) (mm)	
Site		Mean ± SD	Median	Mean ± SD	Median
Mesial	8	1.00 ± 0.07	0.99	0.81 ± 0.08	0.82
Distal	8	1.07 ± 0.13	1.12	0.80 ± 0.077	0.79

Ag^+^	Sample number (*N*)	Bone level before ligature (mm)		Bone level after ligature (day 28) (mm)	
Site		Mean ± SD	Median	Mean ± SD	Median
Mesial	8	0.93 ± 0.19	0.96	0.79 ± 0.24	0.80
Distal	8	0.90 ± 0.20	0.92	0.72 ± 0.24	0.76

In the Ag^+^-coated group, although statistically significant bone loss from baseline was observed on the mesial side at days 14 and 28 (Fig. 3d, e), the magnitude of loss was substantially smaller than that in the control group. On the distal side, no significant bone resorption from baseline was detected at any time point (Fig. 3d, f). After 28 days, Ag^+^-coated implants exhibited approximately 15%–20% bone loss from baseline, which was considerably less than that observed in controls (Table 2). Collectively, these findings indicate that Ag^+^ coating significantly suppressed ligature-induced peri-implant bone resorption.

### Histological analysis

**Fig. 4:**
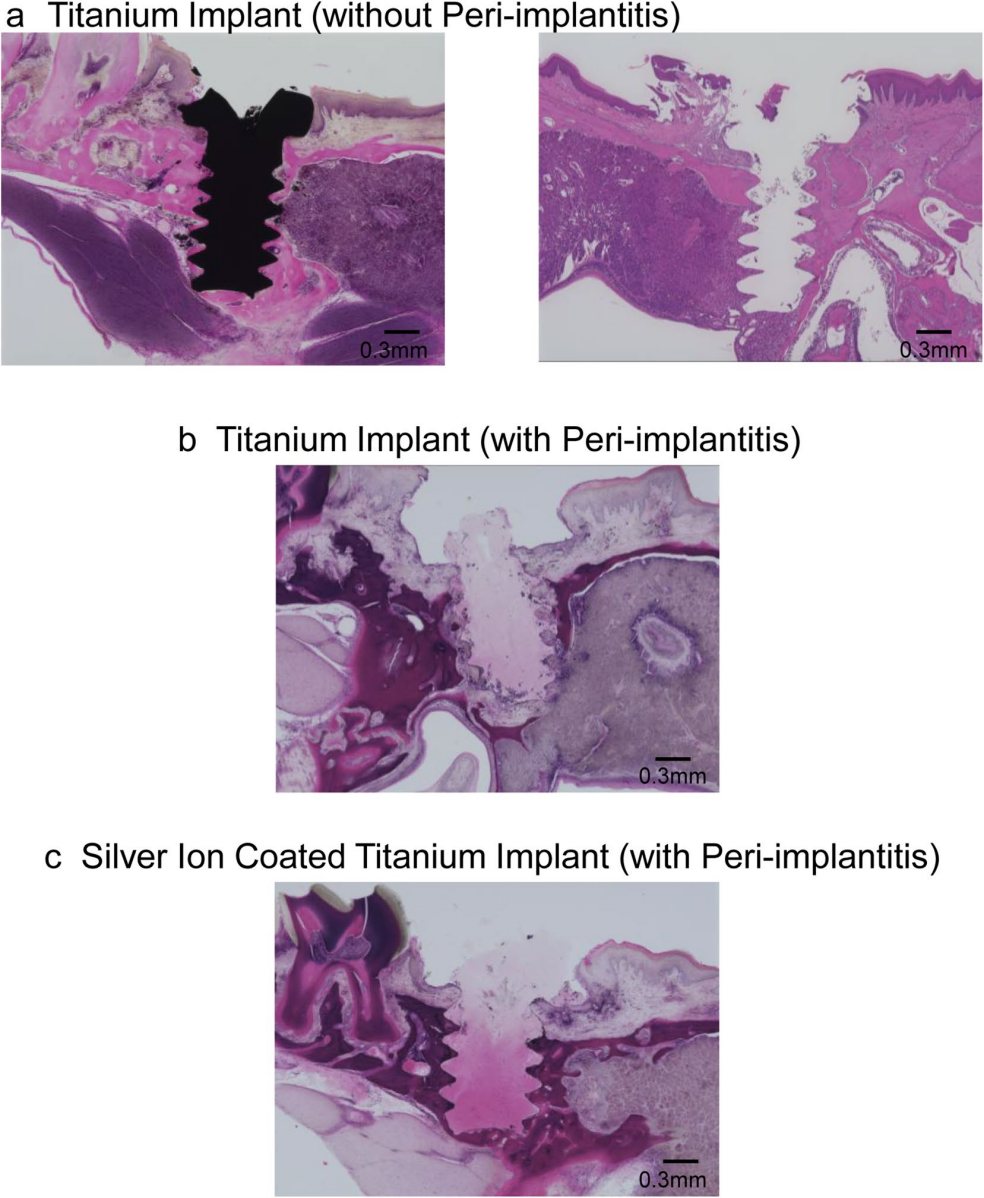
Histological analysis of peri-implant tissues using hematoxylin and eosin staining. (**a**) Negative control group (Ti implant without ligature), exhibiting healthy peri-implant tissues with no evidence of inflammation or bone loss. (**b**) Positive control group (uncoated Ti implant with ligature) showing substantial bone loss and inflammatory cell infiltration. (**c**) Ag^+^-coated implant with ligature, demonstrated reduced bone loss and inflammatory cell infiltration compared with the positive control. Scale bars: 0.3 mm.

The effects of Ag^+^ coating on peri-implant tissues were evaluated using hematoxylin and eosin staining (Fig. 4). Three experimental conditions were examined: (1) negative control—Ti implant placed without ligature, representing healthy peri-implant tissues; (2) positive control—uncoated Ti implant with ligature, representing maximal ligature-induced peri-implantitis; and (3) Ag^+^-coated Ti implant with ligature. The negative control group exhibited healthy peri-implant tissues with no signs of inflammation or bone loss (Fig. 4a). In contrast, the positive control group (uncoated Ti implants with ligature) demonstrated substantial bone loss and inflammatory cell infiltration (Fig. 4b). Notably, Ag^+^-coated implants under ligature-induced peri-implantitis conditions showed significantly reduced bone loss and inflammatory infiltration compared to uncoated Ti implants (Fig. 4c). These histological findings confirm that the Ag^+^ coating effectively attenuates peri-implantitis progression.

### Real-time polymerase chain reaction analysis

**Fig. 5:**
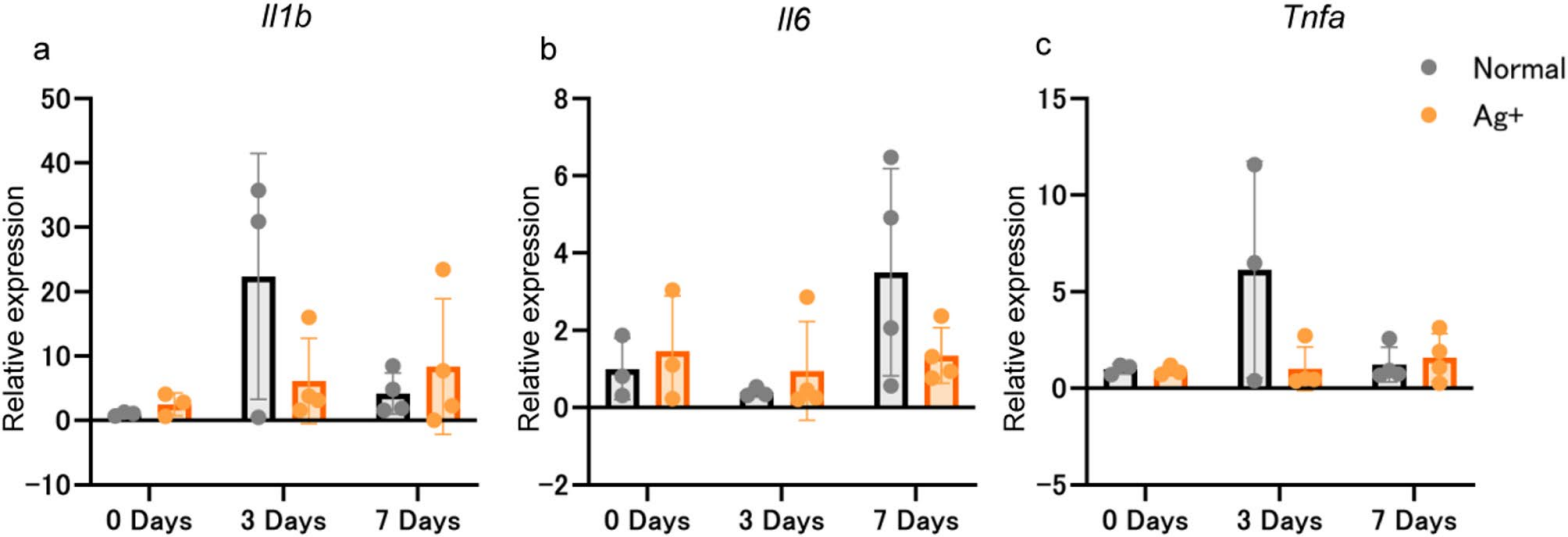
Expression of inflammatory cytokines in peri-implant tissues. Expression of (**a**) *Il1b*, (**b**) *Il6*, and (**c**) *Tnfa* in the control and Ag^+^-coated (Ag^+^) groups was analyzed by real-time PCR at days 0, 3, and 7 post-ligation. Data are presented as mean expression levels relative to β-actin ± standard deviations (*n* = 3–4). No statistically significant differences were detected between the control and Ag^+^-coated groups at any time point (multiple t-tests with Holm-Šídák correction).

Lastly, we analyzed expression levels of the inflammatory cytokines interleukin (IL)-1β, IL-6, and tumor necrosis factor-alpha (TNFα) using real-time polymerase chain reaction (PCR) on days 0, 3, and 7 after ligature placement (Fig. 5). Although no statistically significant differences were observed between the two groups at any time point, the Ag^+^ group tended to show lower expression levels of all three cytokines, particularly IL-1β on days 0 and 3, and TNFα on day 3 (Fig. 5). These findings suggest a trend toward attenuated inflammatory cytokine expression in the Ag^+^-coated group.

## Discussion

### Antibacterial efficacy under anaerobic conditions

This study was designed to evaluate the potential of Ag^+^-coated Ti implants in counteracting *P. gingivalis* infection and suppressing peri-implantitis. Our results, which demonstrated significant in vitro antibacterial activity in the Ti wire configuration against *P. gingivalis*, align with previous research documenting the broad-spectrum antimicrobial effects of Ag^+^^16,17^. Mechanistically, Ag^+^ is known to disrupt bacterial cell membranes, inhibit DNA replication, and induce oxidative stress^19^. The present study extends these findings by demonstrating the efficacy of Ag^+^ coating against *P. gingivalis*, a major pathogen in peri-implantitis^21^, thereby highlighting its potential as a preventive strategy in implant dentistry. It is noteworthy that while the in vitro inhibition zone assay for the screw-type implants did not demonstrate statistically significant antibacterial differences, the in vivo peri-implantitis model revealed significant protective effects. This apparent discrepancy may be attributed to several factors. First, the inhibition zone assay reflects a single time-point measurement of Ag^+^ diffusion under static conditions, whereas the in vivo environment involves continuous Ag^+^ release at the implant–tissue interface over an extended period. Second, the smaller surface area of screw-type implants limits the total amount of Ag^+^ available for diffusion into the agar medium, potentially underestimating the antibacterial capacity in a static assay. Third, in vivo protective effects may also involve modulation of the host inflammatory response beyond direct antibacterial activity, as suggested by the reduced cytokine expression observed in the Ag^+^-coated group.

### Suppression of bone resorption and inflammation

Findings from the ligature-induced peri-implantitis murine model revealed that Ag^+^-coated implants significantly reduced both bone resorption and inflammation compared to uncoated Ti controls. This protective effect was further supported by histological analysis, which showed decreased bone loss and inflammatory cell infiltration around Ag^+^-coated implants. Taken together, these results suggest that Ag^+^ coating is an effective strategy for maintaining peri-implant bone stability—a crucial factor, as untreated peri-implantitis leads to progressive bone destruction and eventual implant failure^4^. These results are consistent with previous murine studies, which demonstrated that bone destruction caused by ligature-induced peri-implantitis is more extensive than that caused by periodontitis, underscoring the need for targeted, implant-specific interventions^26^.

### Clinical implications and advantages over existing therapies

Although the differences in cytokine expression between the Ag^+^-coated and control groups did not reach statistical significance, a trend toward lower IL-1β and TNFα levels was observed in the Ag^+^-coated group. These cytokines are central mediators of peri-implantitis, with elevated levels consistently reported in peri-implant crevicular fluid from affected sites^23–25,27–31^ and correlated with disease severity^24,25^. The observed trend, combined with the significant suppression of bone resorption demonstrated by micro-CT, suggests that Ag^+^ coating may exert a modulatory effect on the local inflammatory response, although further studies with larger sample sizes are needed to confirm this finding. The findings of this study have important clinical implications, particularly in light of the increasing prevalence of peri-implantitis, which affects up to 45% of implant patients^32^. There is a clear need for effective preventive strategies, and the Ag^+^ coating described here offers several advantages over current approaches. First, it delivers localized and sustained antibacterial protection without the need for systemic antibiotics, reducing the risk of antibiotic resistance and related side effects^33^. Second, by preventing bone resorption, it improves long-term implant stability, leading to better patient outcomes and a reduced need for costly and invasive revision procedures. The development of such preventive technologies is particularly timely given the global rise in dental implant use.

### Challenges for clinical translation and long-term safety

Several factors must be addressed to enable clinical translation of Ag^+^-coated implants. First, patient selection criteria should prioritize individuals at high risk of peri-implantitis, such as those with periodontitis, smoking habits, or certain systemic conditions. Second, the long-term safety and biocompatibility of Ag^+^ coatings must be assessed, particularly regarding their impact on osseointegration. Selectively applying the coating only to the transmucosal portion of the implant—where bacterial colonization primarily occurs—may help minimize potential adverse effects on bone integration. Third, the durability of the antibacterial effect should be evaluated to determine appropriate monitoring protocols and the timing of potential reinterventions. Future clinical trials are essential to address these issues and validate the efficacy and safety of Ag^+^-coated implants in humans.

### Future perspectives and study limitations

Taken together, our findings suggest that the Ag^+^ coating technology evaluated in this study demonstrates antibacterial activity against *P. gingivalis* and attenuates inflammation-induced bone loss in a murine ligature-induced peri-implantitis model. While these preclinical results are encouraging, further studies including long-term safety assessments, microbiological analyses, and clinical trials are necessary to establish the translational potential of Ag^+^-coated implants for peri-implantitis prevention.

Nonetheless, this study has several limitations. Although the murine ligature-induced peri-implantitis model provides valuable insights, it does not fully replicate the complexity of human clinical conditions, which involve diverse immune responses, microbial diversity, and mechanical forces. Additionally, microbiological analysis of the ligature-induced peri-implantitis lesions was not performed; therefore, the specific anaerobic bacterial composition at the peri-implant sites remains uncharacterized. Furthermore, quantitative histomorphometric analysis, including bone-to-implant contact (BIC) and peri-implant bone area measurements, was not conducted due to technical limitations associated with the decalcification process required for sectioning of the murine maxilla. Future studies should incorporate undecalcified sections to enable such quantitative assessments. Moreover, the study focused on short-term outcomes (infection and bone loss); further research is needed to assess the long-term stability, biocompatibility, and potential toxicity of Ag^+^ coatings. In particular, the impact of Ag^+^ on initial osseointegration remains unaddressed and should be examined to ensure that its antimicrobial benefits do not compromise implant integration.

Future research should aim to optimize Ag^+^ coatings by fine-tuning ion concentration and release kinetics, explore alternative coating techniques or carrier materials, and validate long-term efficacy in large-animal models and clinical trials. These trials should include clinical, radiographic, microbiological, and patient-reported outcomes, alongside practical assessments of cost-effectiveness and implementation feasibility. Notably, Ag^+^-coated implants offer a passive, localized, and sustained antibacterial barrier—unlike systemic antibiotics or mechanical debridement—representing a potential paradigm shift in peri-implantitis prevention. Combining Ag^+^ coatings with complementary strategies such as surface modifications or localized drug delivery systems may further enhance therapeutic efficacy and broaden clinical utility.

## Methods

### Implant preparation

**Fig. 6:**
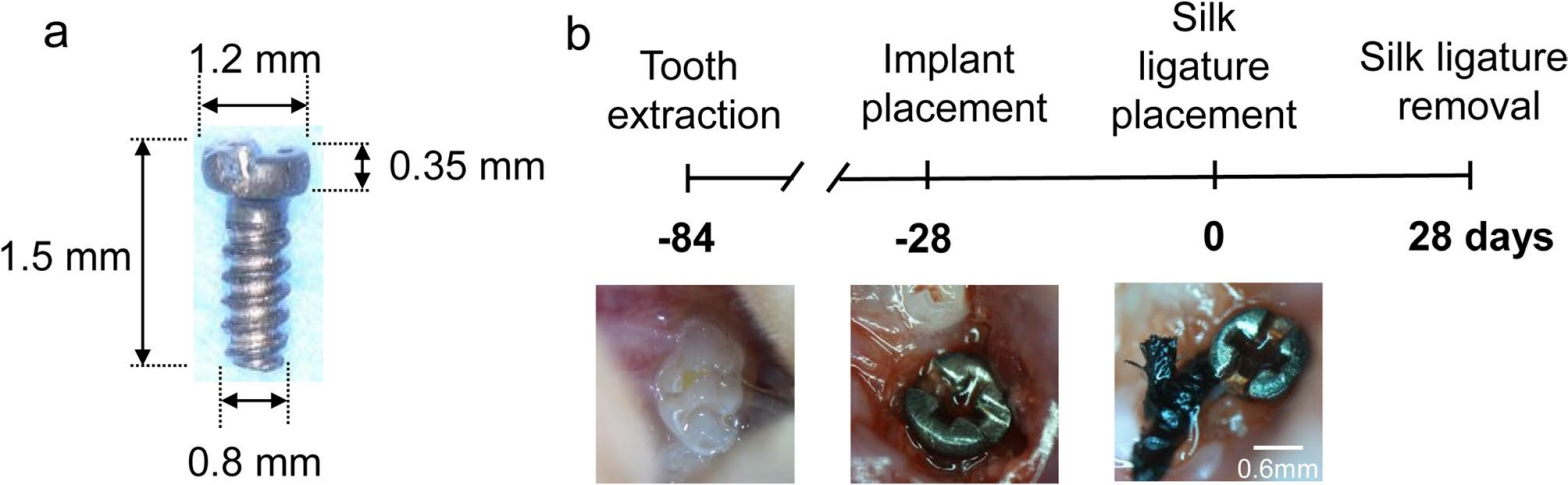
Schematic of implant design and experimental timeline. (**a**) Custom-made pure Ti screw-type implants, with dimensions indicated. (**b**) Overview of the experimental protocol from tooth extraction to euthanasia at designated time points.

Custom-made, pure Ti screw-type dental implants (0.8 mm diameter; 1.5 mm length) were fabricated (Fig. 6a). HAp coating was applied using a soft solution process based on established protocols^34–36^. The resulting HAp-coated Ti implants were then immersed in a 1000 ppm IP6 solution at 50 °C for 24 h to modify the surface and create HAp-IP6-coated Ti specimens. Ag^+^ immobilization was achieved by immersing the HAp-IP6-coated implants in silver nitrate solution (5 and 10 mM, designated Ag^+^(5) and Ag^+^(10), respectively) at 50 °C for 15 min, yielding HAp-IP6-Ag^+^ Ti implants. These Ag^+^ concentrations were selected based on our previous finding that Ag^+^(5) demonstrated effective antibacterial activity on Ti rod specimens^16^. Given the smaller surface area of the screw-type implants compared to rods, which results in a reduced amount of immobilized Ag^+^, the higher concentration [Ag^+^(10)] was also included to ensure sufficient antibacterial efficacy. Unmodified Ti, HAp-coated, and HAp-IP6–coated specimens served as negative controls to isolate the effects of each coating layer.

### Animal model and surgical procedure

All animal procedures were approved by the Institutional Guidelines on Animal Care and Use Committee of Keio University (approval number: A2022-200) and were conducted in accordance with the university’s Institutional Guidelines on Animal Experimentation and the ARRIVE guidelines. A ligature-induced peri-implantitis murine model was employed for the in vivo experiments. In this model, a ligature placed around the implant induces local inflammation and bone loss, effectively simulating the key features of peri-implantitis such as progressive tissue destruction, and is suitable for investigating its distinct pathophysiology compared to periodontitis^26,37^. A total of 152 eight-week-old male C57BL/6 mice (Sankyo Labo Service, Tokyo, Japan) were used in this study. For radiographic and histological analyses, 128 mice were allocated and followed longitudinally with repeated micro-CT imaging at days 0, 7, 14, 21, and 28 post-ligation before being euthanized on day 28. After accounting for attrition due to animal death and implant loss during the healing period, the final sample sizes at the time of ligature placement were: positive control group (uncoated Ti with ligature), *n* = 44; and Ag^+^-coated group (HAp-IP6-Ag^+^(10) with ligature), *n* = 21. However, due to mortality during the experimental period and technical failure of micro-CT imaging, only animals that completed all imaging time points with technically adequate scans were included in the longitudinal analysis (*n* = 8 per group at each time point). For real-time PCR analysis, a separate cohort of 24 mice was used; animals were euthanized at days 0, 3, and 7 post-ligation for peri-implant tissue collection (*n* = 3–4 per group per time point). Mice were housed under specific pathogen-free conditions with a 12-hour light/dark cycle and ad libitum access to sterile water and a standard diet (CLEA Rodent Diet CE-2, Japan).

Anesthesia was induced with 5% isoflurane and maintained at 2.5%–4.0%, followed by subcutaneous or intraperitoneal injection of a triple anesthetic combination: Domitor (0.75 mg/kg), midazolam (4 mg/kg), and Vetorphale (5 mg/kg) at a dose of 0.1 mL per 10 g of body weight. The right maxillary molars were extracted under general anesthesia. After a 56-day healing period, a custom-fabricated Ti screw-type implant was surgically inserted into each healed extraction socket. Twenty-eight days after implantation, peri-implantitis was induced by placing 5 − 0 silk ligatures around the neck of each implant, with the knot positioned on the palatal side. The following experimental groups were defined for the in vivo study: (1) positive control group—uncoated Ti implants with ligature placement; and (2) Ag^+^-coated group—HAp-IP6-Ag^+^(10)-coated Ti implants with ligature placement. For the radiographic cohort, all animals were euthanized on day 28 post-ligation after the final micro-CT scan (Fig. 6b). For the PCR cohort, animals were euthanized at days 0, 3, and 7 post-ligation for tissue collection. At the end of the experimental period, the mice were euthanized by cervical dislocation under deep isoflurane (> 5%) anesthesia. All efforts were made to minimize suffering.

### Bacterial culture and inhibition zone assay

*P. gingivalis* strain W83 (ATCC BAA-308) was obtained from the American Type Culture Collection (Manassas, VA, USA) and provided to our laboratory by JSR Corporation. *P. gingivalis* was cultured in brain heart infusion (BHI) broth supplemented with hemin and menadione (vitamin K) under anaerobic conditions (5% CO₂, 10% H₂, 85% N₂). A 50 µL aliquot of late-log-phase *P. gingivalis* culture (~ 0.3 OD₆₅₀) was uniformly streaked on BHI agar plates supplemented with hemin and menadione using a disposable inoculation loop. The agar consisted of 1.5% (w/v) Bacto™ Agar (Becton Dickinson) in BHI broth. For the inhibition zone assay, various Ti wire and implant screw specimens (as shown in Fig. 1) were placed on *P. gingivalis*–inoculated agar plates and incubated at 37 °C for 48 h under anaerobic conditions. After incubation, the area (mm²) of the inhibition zones surrounding each specimen was measured to quantify antibacterial efficacy.

### Clinical and radiographic analysis

Implant stability was confirmed immediately after placement by verifying the absence of mobility using manual palpation and resistance to rotation with the insertion driver. Clinical signs of inflammation (gingival erythema, swelling, and implant mobility) were assessed at predetermined time points. Peri-implant bone levels were analyzed using micro-CT (R_mCT2, Rigaku Corp., Tokyo, Japan). The peri-implant bone level, defined as the vertical distance from the alveolar bone crest to the apex of the implant (i.e., the length of the implant embedded within bone), was measured at both the mesial and distal aspects (Fig. 2). Bone loss (%) was calculated as: [(Bone level at day 0 − Bone level at day 28) / Bone level at day 0] × 100.

### Quantitative real-time PCR

Peri-implant tissue samples, comprising both soft tissue (peri-implant mucosa) and surrounding hard tissue (alveolar bone), were harvested en bloc using a rongeur-type instrument. Expression levels of three pro-inflammatory cytokines—interleukin-1β (Il1b), interleukin-6 (Il6), and tumor necrosis factor-alpha (Tnfa)—were assessed. Total RNA was extracted from these combined tissue samples using TRIzol reagent (Invitrogen, Carlsbad, CA, USA) and the RNeasy Mini Kit (QIAGEN, Antwerp, Belgium) and stored at − 80 °C. First-strand cDNA synthesis was performed using oligo (dT) primers and reverse transcriptase (Wako Pure Chemicals Industries) following established protocols^38^. Quantitative real-time PCR was conducted using SYBR Premix ExTaq II reagent on a CFX Connect Real-Time PCR Detection System (Bio-Rad Laboratories, Hercules, CA, USA) following the manufacturer’s guidelines. β-actin expression served as an internal control. The following primers were used:

*β-actin* forward: 5′-TGAGAGGGAAATCGTGCGTGAC-3′.

*β-actin* reverse: 5′-AAGAAGGAAGGCTGGAAAAGAG-3′.

*Tnfa* forward: 5′-AAGCCTGTAGCCCACGTCGT-3′.

*Tnfa* reverse: 5′-GGCACCACTAGTTGGTTGTCTTTG-3′.

*Il1b* forward: 5′-AAGTTGACGGACCCCAAAAGAT-3′.

*Il1b* reverse: 5′-AGCTCTTGTTGATGTGCTGCTG-3′.

*Il6* forward: 5′-GTCCTTAGCCACTCCTTCTG-3′.

*Il6* reverse: 5′-CAAAGCCAGAGTCCTTCAGAG-3′.

### Statistical analysis

Data are expressed as mean ± standard deviation (S.D.). For the inhibition zone assay (Fig. 1), differences among multiple groups were analyzed using one-way analysis of variance (ANOVA) followed by Tukey’s post-hoc test. For longitudinal micro-CT bone level data (Fig. 3), repeated measures one-way ANOVA followed by Dunnett’s post-hoc test was used to compare each time point against baseline (day 0). For cytokine expression data (Fig. 5), multiple t-tests with Holm-Šídák correction were used to compare the control and Ag^+^-coated groups at each time point. All statistical analyses were performed using GraphPad Prism 10 (GraphPad Software, San Diego, CA, USA). Statistical significance was denoted as follows: *p < 0.05; **p < 0.01; ***p < 0.001; n.s., not significant). Effect sizes for pairwise comparisons were calculated using Cohen’s d with SPSS Version 25 (IBM Corp., Armonk, NY, USA), with values of 0.2, 0.5, and 0.8 interpreted as small, medium, and large effects, respectively. A *p*-value < 0.05 was considered statistically significant.

## Data Availability

The data supporting the findings of this study are available from the corresponding author upon reasonable request.
